# Cross-cultural validation of the Cebuano version of a screening questionnaire for Parkinson's disease

**DOI:** 10.1055/s-0042-1758652

**Published:** 2022-12-29

**Authors:** Daryl Dakay, Faith Tangcalagan, Emilio Villanueva III, Joshua Abejero, Gerard Saranza, on behalf of the Philippine Parkinson's disease Prevalence Study Group

**Affiliations:** 1 Chong Hua Hospital Mandaue, Department of Internal Medicine, Cebu, Philippines.; 2 University of the Philippines, Department of Pathology, Manila, Philippines.; 3 Chong Hua Hospital, Department of Internal Medicine, Section of Neurology, Cebu, Philippines.

**Keywords:** Parkinson Disease, Philippines, Epidemiology, Doença de Parkinson, Filipinas, Epidemiologia

## Abstract

**Background** In the Philippines, the exact prevalence of Parkinson's disease (PD) has not yet been determined. Although cases can be extrapolated from medical registries, this method may undermine actual case rates. A reliable screening tool for PD is essential for a timely diagnosis and community-based epidemiological studies. The most widely used screening questionnaire for PD diagnosis was developed by Tanner et al. *,* which consists of nine questions about the motor symptoms of PD. Although this questionnaire has been translated to several languages, the translated version must be validated for use in our local setting.

**Objective** To determine the validity of the Cebuano version of a PD screening questionnaire.

**Method** The questionnaire was translated from English to Cebuano by a hired language specialist. Each item was supplied with a *yes* , *no* , or *don't know* answer. A total of 73 patients with PD and 244 control subjects completed the study.

**Results** The overall Cronbach alpha for internal consistency of the questionnaire was 0.9410. The item on *tremor* had the highest sensitivity (97.26%), while the item on *problems with buttoning* had the highest specificity (100.00%). A cut-off score ≥ 3 obtained the best Youden index (99.18%), with a sensitivity of 100.00% and a specificity of 99.18%. The questionnaire had an almost perfect predictive ability to diagnose PD (AUC of 0.9994).

**Conclusion** The translated version of the Tanner questionnaire is a validated instrument to identify PD in a literate Cebuano population.

## Resumo

**Antedecentes** Nas Filipinas, a prevalência exata da doença de Parkinson (DP) ainda não foi determinada. Embora os casos possam ser extrapolados dos registros médicos, esse método pode prejudicar as taxas reais de casos. Uma ferramenta de triagem confiável para DP é essencial para um diagnóstico oportuno e estudos epidemiológicos baseados na comunidade. O questionário de triagem mais utilizado para o diagnóstico da DP foi desenvolvido por Tanner et al., que consiste em nove questões sobre os sintomas motores da DP. Embora este questionário tenha sido traduzido para vários idiomas, a versão traduzida deve ser validada para uso em nosso meio.

**Objetivo** Determinar a validade da versão Cebuano de um questionário de triagem de DP.

**Métodos** O questionário foi traduzido do inglês para o cebuano por um especialista em idiomas contratado. Cada item foi fornecido com uma resposta “sim”, “não” ou “não sei”. Um total de 73 pacientes com DP e 244 controles completaram o estudo.

**Resultados** Em geral o alfa de Cronbach para consistência interna do questionário foi de 0,9410. O item “tremor” teve a maior sensibilidade (97,26%), enquanto o item “problemas com abotoamento” teve a maior especificidade (100,00%). Um escore de corte > 3 obteve o melhor índice de Youden (99,18%), com sensibilidade de 100,00% e especificidade de 99,18%. O questionário teve uma capacidade preditiva quase perfeita para diagnosticar DP (AUC de 0,9994).

**Conclusão** A versão traduzida do questionário de Tanner é um instrumento validado para identificar a DP em uma população cebuana alfabetizada.

## INTRODUCTION

Parkinson's disease (PD) is a chronic and progressive neurodegenerative disorder characterized by motor and non-motor features. The diagnosis of PD remains clinical, based primarily on demonstrating its cardinal motor symptoms. 1 In the Philippines, the prevalence of parkinsonism is estimated to be less than 1% in a 2007 study conducted by the Philippine Neurological Association. 2 This study used a 10-item questionnaire to determine the prevalence of several neurological conditions, including stroke, dementia, migraine, and epilepsy; there were only two items related to parkinsonism (i.e., a question on tremor and bradykinesia). Moreover, the diagnosis is often delayed due to financial constraints, and misconceptions about parkinsonian symptoms as manifestations of normal aging or the effects of excessive working (locally termed as “ *pasma* ”). As the disease progresses, the symptoms cause significant impairment of function and quality of life, prompting the need for treatment. 3 Therefore, having a sensitive, specific, and simple screening tool is essential in the timely detection and management of PD. The questionnaire can also facilitate epidemiological studies in PD. There have been several screening instruments designed to detect parkinsonism and PD. 4 The most widely used screening questionnaire was developed by Tanner et al., 5 which has been translated to several other languages *.* The present study aimed to investigate the validity of the Cebuano version of the Tanner questionnaire.

## METHODS

### Study design

This cross-sectional validation study included patients clinically diagnosed with PD and controls. Permission for translation of the original questionnaire was obtained by one of the authors (G. S.). The questionnaire consists of nine individual questions about the motor symptoms of PD, including problems with agility, balance, and hand dexterity, micrographia, gait abnormalities (e.g., shuffling and freezing of gait), tremors, hypomimia, and hypophonia. The questionnaire was translated from English to Cebuano by a hired English-Cebuano language specialist with particular attention to local idiomatic expression for cross-cultural adaptation. It was then independently back translated to English by a second bilingual person. The final version of the questionnaire accounted for syntax uniformity and any differences between the original and retranslated versions ( Table 1 ). Questions were supplied with *yes* , *no,* and *don't know* answers by the study participants. *Don't know* responses were considered negative for the computation of sensitivity and specificity. The study was conducted in accordance with the Declaration of Helsinki for biomedical research involving human subjects. It was approved by the Chong Hua Hospital Institutional Review Board (Protocol No. 4921-05). Coronavirus disease precautionary measures were observed during the data collection.

**Table 1:** Tanner questionnaire for Parkinson's disease

Item No.	English version	Cebuano version	Response		
1	Do you have trouble arising from a chair?	*Maglisod ba ka pagbarog gikan maglingkod?*	Yes/ *Oo*	No/ *Dili*	Don't know/ *Wala kabalo*
2	Is your handwriting smaller than it once was?	*Ang imong agi sa pagsulat karon, mas gagmay ba kaysa kaniadto?*	Yes/ *Oo*	No/ *Dili*	Don't know/ *Wala kabalo*
3	Do people tell you that your voice is softer than it once was?	*Naa bay misulti nimo nga ang imo karong tingog mas hinay kaysa kaniadto?*	Yes/ *Oo*	No/ *Dili*	Don't know/ *Wala kabalo*
4	Is your balance poor when walking?	*Aduna ka bay problema sa pagbalanse samtang maglakaw?*	Yes/ *Oo*	No/ *Dili*	Don't know/ *Wala kabalo*
5	Do you feel you suddenly seem to freeze in doorways?	*Aduna bay higayon nga kalit ka lang dili ka makalihok inig agi nimo sa pultahan?*	Yes/ *Oo*	No/ *Dili*	Don't know/ *Wala kabalo*
6	Does your face seem less expressive than it used to?	*Dili na ba madayagon ang imong panagway kay sa imong naandan nga bayhon?*	Yes/ *Oo*	No/ *Dili*	Don't know/ *Wala kabalo*
7	Do your arms and legs shake?	*Mangurog ba ang imong mga kamot ug paa?*	Yes/ *Oo*	No/ *Dili*	Don't know/ *Wala kabalo*
8	Do you have trouble doing buttons?	*Maglisod ba ka mamatones?*	Yes/ *Oo*	No/ *Dili*	Don't know/ *Wala kabalo*
9	Do you shuffle your feet and take tiny steps when you walk?	*Magsaguyod ba imong tiil ug mugbo na ang imong lakang sa paglakaw?*	Yes/ *Oo*	No/ *Dili*	Don't know/ *Wala kabalo*

### Setting and participants

The sample size was computed using the pROC version 1.17.0.1 package of R version 4.0.3. A minimum sample size of 307 participants distributed in 1:4 ratio (i.e., 62 participants with PD and 244 control subjects) would achieve 80% power with 5% significance level in a receiver operating characteristic (ROC) curve analysis. This minimum sample size would be able to detect a significant area under the curve (AUC) of the translated questionnaire in diagnosing PD with 10% one-sided margin of AUC from the null (AUC = 0.50). The authors have carefully selected and successfully recruited 73 PD patients and 244 controls from the hospital database of Chong Hua Hospital and the private clinics of the two authors (J. A. and G. S.). The overall power analysis using the same R package showed that with this sample size, the study achieved 100% power. All patients were diagnosed with idiopathic PD according to the United Kingdom Brain Bank Criteria. 6 Parkinson disease patients (1) with severe communication and cognitive impairment, (2) who are unable to participate in everyday activities due to other serious co-morbidities, (3) with an age of onset of less than 40 years old, and (4) with secondary causes of parkinsonism having been excluded. Caregivers were allowed to assist the patients in completing the questionnaire to enable patients with disabling motor impairment to participate.

### Data collection

To observe physical distancing measures, the majority of the patients were contacted through their mobile phones from June to October 2021. Informed consent, available both in English and Cebuano, was obtained before administering the questionnaire. The questionnaire was electronically sent through different platforms depending on the patient's preference. Other PD patients were asked to participate during their clinic visits with their attending neurologist (J.A. and G.S.). The patients were instructed to answer the questions based on their current state and not on their previous condition (i.e., disease onset or before starting medications). Demographics and information about the age at onset, Hoehn and Yahr (H&Y) stage and their PD medications were also collected. The daily dose of the medications was computed using the levodopa equivalent dose conversion factors. 7

The specificity of the questionnaire was ascertained in a population of 244 randomly selected patients attending the outpatient clinics. These patients were more than 40 years old and sought medical attention for non-neurological reasons. Informed consent was also obtained before the translated questionnaire was administered. All patients were evaluated for tremors, rigidity, bradykinesia, masked facies, gait disturbance, and postural stability according to the Movement Disorder Society-sponsored revision of the Unified Parkinson Disease Rating Scale (MDS-UPDRS). 8 The screening physicians (D. D. and F. T.) underwent training with the MDS-UPDRS. Regardless of the scores on the translated screening questionnaire, participants who were suspected of having parkinsonism were advised to consult (without any fee) the two neurologists (J. A. and G. S.) who are experienced in the diagnosis of PD.

### Data analyses

For the internal consistency analysis, we calculated the Cronbach alpha for the screening questionnaire. The sensitivity, specificity, and *don't know* rates were determined for each question. The differences between the PD and control subjects for each item were determined using the chi-squared test. A score of 0 is assigned for each question with a negative answer and 1 with a positive answer. *Don't know* answers were considered negative answers and given a score of 0. The total scores for each question were computed between two groups using the Mann-Whitney U test. The performance of the total score was analyzed and assessed. The optimal cut-off score was determined to distinguish PD from control subjects employing a ROC curve analysis.

## RESULTS

Seventy-five PD patients were enrolled in the study ( Table 2 ), but only 73 completed the questionnaire. There were 35 male and 38 female patients, with ages ranging from 44 to 85 years. The median disease duration was 4 years, with most patients belonging to H&Y stages II and III. The median daily levodopa dose was computed to be at 625 mg/day. Five PD patients were treatment naïve and were only recently diagnosed with PD. A total of 244 patients were included in the control group, consisting of 145 males and 99 females, with ages ranging from 40 to 76 years. After careful evaluation, none of them was deemed to have parkinsonism or PD. There was no significant difference between the number of PD patients and controls with low (high school and below) and high level (college/university and beyond) educational attainment ( *p* = 0.182, chi-squared test of homogeneity).

**Table 2:** Demographic and general characteristics of patients with Parkinson's disease and of the control group

		Parkinson's disease	Control group
n		73	244
Sex (male: female)		35:38	145:99
Age (median [IQR])		66 (15)	48 (13)
Educational level (n [%])	Elementary	17 (23%)	33 (14%)
	Secondary	26 (36%)	89 (36%)
	College/University	27 (37%)	119 (49%)
	Postgraduate	3 (4%)	3 (1%)
Disease duration, years (median [IQR])		4 (4)	–
Levodopa equivalent dosage, mg/day (median [IQR])		625 (645)	–
Hoehn and Yahr Stage (n [%])	I	9 (12%)	–
	II	17 (23%)	–
	III	29 (40%)	–
	IV	13 (18%)	–
	V	5 (7%)	–

Abbreviation: IQR, interquartile range.

The overall Cronbach alpha for internal consistency of the questionnaire was 0.9410 ( Supplementary table ). Under conditions in which each question was individually deleted, the Cronbach alpha showed similar coefficients of consistency. The *don't know* response rate, sensitivity, and specificity of the nine individual symptom questions are shown in Table 3 . All items showed significant differences between PD and control subjects and were valid to distinguish the two groups ( *p* < 0.001, chi-squared test). The *don't know* response rate was the highest for the 6 ^th^ question on *less expressive face* . The symptom question with the highest sensitivity was item 7 (97.26%), concerning *shaking with arms and legs* . Question 8 (“problems with buttoning”) had the highest specificity (100.00%). The total score of the PD group (median = 6; IQR = 3) was significantly higher than that of the control subjects (median = 0, interquartile range [IQR] = 0; *p* < 0.001, Mann-Whitney U test).

**Table 3:** The sensitivity and specificity of individual items of the Cebuano version of the Parkinson's disease questionnaire

Question	*Don't know* response		Positive		Negative		Sensitivity (%)	Specificity (%)	*P* -value*
	n	%	True	False	True	False			
**1**	**0**	**-**	**44**	**2**	**242**	**29**	**60.27%**	**99.18%**	**< 0.001**
2	1	0.32%	54	7	237	19	73.97%	97.13%	< 0.001
3	0	–	50	2	242	23	68.49%	99.18%	< 0.001
4	0	–	58	2	242	15	79.45%	99.18%	< 0.001
5	0	–	44	1	243	29	60.27%	99.59%	<0.001
6	2	0.63%	45	3	241	28	61.64%	98.77%	< 0.001
7	0	–	71	12	232	2	97.26%	95.08%	< 0.001
8	0	–	49	0	244	24	67.12%	100.00%	< 0.001
9	0	–	46	2	242	27	63.01%	99.18%	< 0.001

Note: *Chi-squared test.

**Figure 1:**
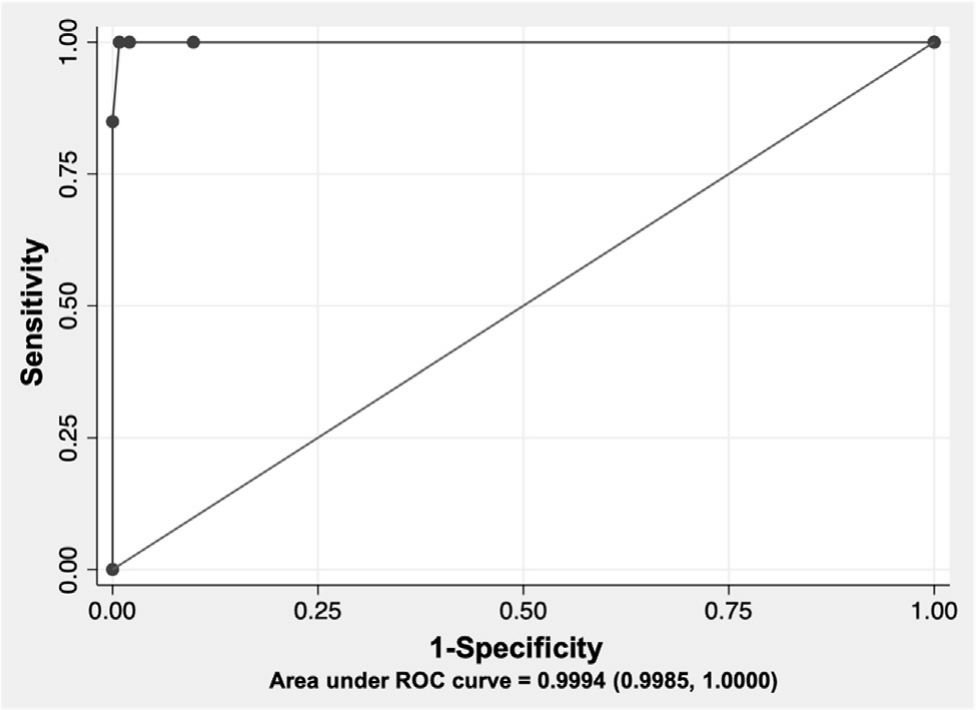
Receiver operating characteristic (ROC) curve for the total score of the Cebuano version of the Parkinson's disease questionnaire.

Table 4 shows the sensitivity, specificity, and Youden index of the total score of the questionnaire at each cut-off point. For example, a cut-off of 3 means that participants who responded positively to three or more symptom questions were considered *positive* to the test (i.e., likely to have PD). A cut-off of 3 obtained the best Youden index (99.18%), with a sensitivity of 100.00% and a specificity of 99.18%. The questionnaire had an almost perfect predictive ability to diagnose PD, as shown by the ROC curve (AUC = 0.9994) ( Figure 1 ).

**Table 4:** The sensitivity and specificity of the total score of the Cebuano version of the Parkinson's disease questionnaire

Cut-off score	Positive				Negative				Sensitivity (%)	Specificity (%)	Youden index (%)
	True	False	n	%	True	False	n	%			
1	73	24	97	30.60%	220	0	220	69.40%	100.00%	90.16%	90.16%
2	73	5	78	24.61%	239	0	239	75.39%	100.00%	97.95%	97.95%
3	73	2	75	23.66%	242	0	242	76.34%	100.00%	99.18%	99.18%
4	62	0	62	19.56%	244	11	255	80.44%	84.93%	100.00%	84.93%
5	56	0	56	17.67%	244	17	261	82.33%	76.71%	100.00%	76.71%
6	49	0	49	15.46%	244	24	268	84.54%	67.12%	100.00%	67.12%
7	34	0	34	10.73%	244	29	283	89.27%	46.58%	100.00%	46.58%
8	26	0	26	8.20%	244	47	291	91.80%	35.62%	100.00%	35.62%
9	15	0	15	4.73%	244	58	302	95.27%	20.55%	100.00%	20.55%

## DISCUSSION

To date, the exact prevalence of PD in the Philippines has not yet been established. A previous study, in 2007, estimated the prevalence of parkinsonism in the Philippines to be lower than 1%. 2 However, there was no case ascertainment, and mimics of PD may have been included. 9 Although the incidence and prevalence rate of PD can be extrapolated from hospital databases, this method may underestimate actual case rates. Community cases are usually missed because of the lack of appropriate data collection instruments. Adding to the challenge is the linguistic diversity in the Philippines, with more than 100 spoken languages and dialects. Filipino and Cebuano are the two most spoken languages in the Philippines, with the latter used in the southern part of the Philippines. This makes our questionnaire more valuable as it applies to a broader scale of the Filipino population, which can later be used in a nationwide prevalence study on PD. Considering that not all patients with PD are tremor-predominant, our questionnaire, which consists of nine questions on the cardinal motor symptoms of PD, will be more sensitive in detecting the disease in the community setting.

Under certain conditions wherein an item in the questionnaire is not applicable, the translated questionnaire remained valid since the overall Cronbach alpha for internal consistency was > 0.7. For instance, a mute person can still answer the questionnaire without affecting its validity even if item 3 is omitted. To differentiate PD patients from unaffected individuals, a cut-off score of ≥ 3 was chosen since this obtained the best Youden index, with high sensitivity and sensitivity (100% and 99.18%, respectively), making it a reliable screening tool. This means that an individual who responds to the questionnaire with at least three positive answers is likely to have PD. There was no PD patient in our study who had a score < 3, even if our cohort included patients in the early stages of the disease (i.e., H&Y stages I and II) and patients who claimed to have well-controlled symptoms. The cut-off score in our study was similar to that of the original (5) and the other translated versions of the questionnaire (10). Moreover, the question on *shaking of the arms and legs* attained the highest sensitivity but also had the lowest specificity among all items. Tremor is the most common presenting symptom of PD, but it can also be a symptom of other neurological disorders, such as essential tremor. The item on *problems with buttoning* , suggesting problems with hand dexterity, had the highest specificity, similar to the other versions of the questionnaire. 10 11 Of note, the item on *less expressive face* had the highest number of *don't know* responses. This is contrary to findings of other translation studies 10 11 and may reflect either the lack of awareness of this feature as a symptom of PD or its inconspicuousness because of the frequent use of face mask during these times. 12 Moreover, the ROC curve of our translated questionnaire further supports its reliability in distinguishing PD from controls.

Some factors may affect the results of our study. The PD group may differ from community-based cohorts concerning sex predilection and degree of disability. Interestingly, there were more female participants in the PD group (male: female ratio of 1:1.1), although the sex ratio between the PD and control group did not significantly differ ( *p* = 0.082, chi-squared test of homogeneity). This finding, however, corroborates the observation of previous studies, which showed a slight female predominance of parkinsonism in the Philippines, 13 unlike in other populations where PD is generally known to affect males more than females. 14 Further studies are needed to confirm this finding. Moreover, literacy is a critical factor in answering the questionnaire; however, our study population contained a similar proportion of individuals with a low and high educational attainment. Disease severity is also a potential factor, especially if there is an insufficient number of participants in the early and late stages, resulting in a falsely high sensitivity. Our study included individuals with PD in different H&Y stages, including treatment-naïve patients and recently diagnosed cases.

This study is not without any limitations. Since the majority of PD patients in our study were identified through the hospital database, we relied on the clinical diagnosis of different attending neurologists. We did not have access to their previous records and diagnostic work-up; thus, our cohort may include patients with other forms of parkinsonism. Nonetheless, the Tanner questionnaire was developed to screen for parkinsonism and not just PD. 5 Moreover, cognitive impairment as an exclusion criterion was only assessed subjectively; no formal cognitive testing was done. For some patients whose H&Y stages were not indicated in their medical records, only a crude assessment was done through teleconsultation if they could not visit the hospital given the pandemic restrictions.

In conclusion, the translated version of the Tanner questionnaire is a validated instrument to identify PD in a literate Cebuano population. A Filipino version of this questionnaire is also currently in development. These screening tools will be used in future epidemiological studies, particularly in establishing the prevalence of parkinsonism and PD in the Philippines.
