# Supplementary material for: Cross-cultural validation of the Cebuano version of a screening questionnaire for Parkinson's disease
Source: Arq Neuropsiquiatr. 2022 Dec 29;80(12):1239–45. doi: 10.1055/s-0042-1758652 (PMC9800160; doi:10.1055/s-0042-1758652)
Supplement: Supplementary file 1 — Supplementary Material [file 10-1055-s-0042-1758652-s220018.pdf]

Supplementary table Internal consistency of the screening questionnaire

Question	Significance of Cronbach alpha if the question was deleted
1	0.9342
2	0.9382
3	0.9366
4	0.9282
5	0.9354
6	0.9397
7	0.9311
8	0.9309
9	0.9318
Overall Cronbach alpha	0.9410

 © 2022. Academia Brasileira de Neurologia. All rights reserved.
